# "Nested" cryptic diversity in a widespread marine ecosystem engineer: a challenge for detecting biological invasions

**DOI:** 10.1186/1471-2148-11-176

**Published:** 2011-06-21

**Authors:** Peter R Teske, Marc Rius, Christopher D McQuaid, Craig A Styan, Maxine P Piggott, Saïd Benhissoune, Claudio Fuentes-Grünewald, Kathy Walls, Mike Page, Catherine RM Attard, Georgina M Cooke, Claire F McClusky, Sam C Banks, Nigel P Barker, Luciano B Beheregaray

**Affiliations:** 1 Molecular Ecology Laboratory, School of Biological Sciences, Flinders University, Adelaide, SA 5001, Australia; 2 Molecular Ecology Laboratory, Department of Biological Sciences, Macquarie University, Sydney 2109, Australia; 3 Department of Zoology and Entomology, Rhodes University, Grahamstown 6140, South Africa; 4 Molecular Ecology and Systematics Group, Rhodes University, Grahamstown 6140, South Africa; 5 Centre for Invasion Biology, Zoology Department, University of Cape Town, Rondebosch 7701, South Africa; 6 Current Address: Department of Evolution and Ecology, University of California, One Shields Avenue, Davis, California 95616, USA; 7 School of Life and Environmental Sciences, Deakin University, PO Box 423, Warrnambool, Victoria 3280, Australia; 8 Centre for Ocean Studies, The University of Western Australia, 35 Stirling Highway, Crawley, Perth, Western Australia 6009, Australia; 9 Fenner School of Environment and Society, Australian National University, Canberra, Australian Capital Territory 0200, Australia; 10 Laboratoire des Substances Naturelles, Equipe d'Océanographie Biologique, Département de Biologie, Faculté des Sciences, BP.403, Agadir principale, 80.000, Agadir, Morocco; 11 Institut de Ciències del Mar, Consejo Superior de Investigaciones Científicas (ICM-CSIC), Passeig Maritím de la Barceloneta 37-49, 08003, Barcelona, Spain; 12 Environmental and Marine Response, Biosecurity Response | Post-Border, MAF Biosecurity New Zealand, Ministry of Agriculture and Forestry, Te Manatu Ahuwhenua, Ngaherehere, New Zealand; 13 National Institute of Water and Atmospheric Research (NIWA), PO Box 893, Nelson 7040, New Zealand

## Abstract

**Background:**

Ecosystem engineers facilitate habitat formation and enhance biodiversity, but when they become invasive, they present a critical threat to native communities because they can drastically alter the receiving habitat. Management of such species thus needs to be a priority, but the poorly resolved taxonomy of many ecosystem engineers represents a major obstacle to correctly identifying them as being either native or introduced. We address this dilemma by studying the sea squirt *Pyura stolonifera*, an important ecosystem engineer that dominates coastal communities particularly in the southern hemisphere. Using DNA sequence data from four independently evolving loci, we aimed to determine levels of cryptic diversity, the invasive or native status of each regional population, and the most appropriate sampling design for identifying the geographic ranges of each evolutionary unit.

**Results:**

Extensive sampling in Africa, Australasia and South America revealed the existence of "nested" levels of cryptic diversity, in which at least five distinct species can be further subdivided into smaller-scale genetic lineages. The ranges of several evolutionary units are limited by well-documented biogeographic disjunctions. Evidence for both cryptic native diversity and the existence of invasive populations allows us to considerably refine our view of the native versus introduced status of the evolutionary units within *Pyura stolonifera *in the different coastal communities they dominate.

**Conclusions:**

This study illustrates the degree of taxonomic complexity that can exist within widespread species for which there is little taxonomic expertise, and it highlights the challenges involved in distinguishing between indigenous and introduced populations. The fact that multiple genetic lineages can be native to a single geographic region indicates that it is imperative to obtain samples from as many different habitat types and biotic zones as possible when attempting to identify the source region of a putative invader. "Nested" cryptic diversity, and the difficulties in correctly identifying invasive species that arise from it, represent a major challenge for managing biodiversity.

## Background

Biological invasions are a major global threat that can fundamentally and irreversibly modify native communities [1,2]. Particularly when a biological invasion involves an ecosystem engineer, the consequences for an invaded ecosystem can be catastrophic [3]. Ecosystem engineers monopolise space, accumulate biomass and have strong effects on species interactions by increasing architectural complexity of ecosystems and moderating environmental extremes [4]. Non-indigenous species that function as ecosystem engineers are of major concern because they can replace indigenous habitat-forming species [5,6] and drastically alter an invaded habitat [7,8]. To maintain the diversity and integrity of biotic habitats, it is thus of great importance that such species are correctly identified and managed.

Coastal environments are among the most threatened ecosystems, with invasions of coastal assemblages across and between oceans facilitated by the movement of ocean-going ships and aquaculture [9-11]. However, in many of the world's coastal regions, a large proportion of marine species cannot be clearly identified as being either native or introduced due to a lack of systematic, biogeographic and historical evidence [12]. The increasing availability of DNA sequence data has improved this situation to some extent, resulting in an exponential increase in the identification of cryptic biodiversity [13]. Particularly in the case of poorly studied marine invertebrate groups, genetic methods can enable researchers to differentiate between recently introduced exotic species that should be monitored and controlled, and long-established, cryptic species that may have been previously overlooked and that may even require protection.

The ascidians (Chordata: Urochordata) are a group of sessile, filter feeding marine invertebrates that include both important ecosystem engineers and aggressively invasive species [14,15]. Many ascidians are major occupiers of primary space along temperate coasts, where they provide habitat for numerous other organisms [16,17] by enhancing habitat complexity when aggregated [18]. Although ascidians have low natural dispersal potential because their lecithotrophic larvae remain in the plankton for very short periods of time (minutes to hours in most species) [19,20], several species are recognised as pests on a global scale, occurring on multiple continents [21-23]. Dispersal on smaller scales may occur naturally as larvae attach themselves to floating objects that are moved around by currents, but adults attached to vessel hulls are considered to be the most likely vectors facilitating the worldwide spread of these species [24-26]. As for many other marine invertebrate groups, the taxonomy of some ascidians is poorly resolved [22,27], and recent genetic studies have indicated that several supposedly cosmopolitan species are in fact comprised of two or more genetic lineages that should be treated as distinct species [28-32].

Here we examine the large, solitary ascidian *Pyura stolonifera *(Heller, 1878), which is an important foundation species particularly in temperate coastal regions of the southern hemisphere [16,18,33]. The taxonomic status and origin of the species are unclear, and have generated an extensive debate [34-38]. It remains uncertain whether populations in Africa, Australasia and South America are the fragmented remains of a pan-Gondwanan species [35,36] or whether the species originated in one region and was recently been introduced to the other two regions [34,39]. It is also disputed whether *P. stolonifera *is a single species [36] or a species complex [34]. We study genetic patterns between regional populations to determine a) levels of cryptic diversity, b) the invasive or native status of each regional population and c) the most appropriate sampling design for identifying the boundaries of each evolutionary unit. Our findings indicate the presence of multiple genetic lineages within regions, which, together with inadequate sampling, can seriously hinder our capacity to detect invasive populations.

## Methods

### Study taxon

*Pyura stolonifera *is particularly common in southern Africa and Australia [35], but localised populations have also been reported from South America [16,40], northwestern Africa [37,38] and, most recently, New Zealand [39]. The taxon is an important ecosystem engineer that dominates intertidal and subtidal habitats in Africa [17,37,41], Australia and New Zealand [39], and intertidal areas in Chile [15], where it achieves among the highest biomasses ever reported in such environments [16]. *Pyura stolonifera *forms extremely large aggregations, resulting in aggressive monopolisation of the available substratum [15].

### Sampling and amplification of genetic markers

A total of 518 ingroup samples were collected in all regions from which there are reliable reports of *Pyura stolonifera*, except Senegal. Within each region, samples were collected at several sites that span the taxon's entire range, including 16 sites in Africa, 26 sites in Australia, seven sites in New Zealand and one site in Chile (Table 1). A small piece of mantle tissue (< 1 cm^3^) from each individual sampled was preserved in a solution containing 70% ethanol and 30% TE buffer. This medium was replaced on a daily basis until it no longer changed color and until the tissue had become completely white. Obtaining high quality DNA proved difficult, and even an extraction protocol developed to eliminate contaminants present in ascidian tissues and tested specifically on *Pyura stolonifera *[42] did not produce better results than standard extraction protocols. We consequently used a salting-out protocol to extract DNA [43].

**Table 1:** Sample localities and number of sequences generated for four different markers

Region	Site	**Site No**.	GPS coordinates	COI	ANT	ATPSα	18S
South Africa							
SW	Langebaan	1	33°01'07''S, 17°56'48''E	7	16	3	3
SW	Yzerfontein	2	33°20'49''S, 18°09'06''E	13	26	2	2
SW	False Bay	3	34°07'14''S, 18°27'31''E	1	6	1	1
S	Mossel Bay	4	34°10'42''S, 22°08'41''E	1	0	1	1
S	Knysna	5	34°03'17''S, 23°03'46''E	4	10	3	3
S	Plettenberg Bay	6	34°05'56''S 23°22'45''E	4	0	0	0
S	Tsitsikamma	7	33°58'52''S 23°38'32''E	2	0	0	0
S	Port Elizabeth	8	33°57'59''S, 25°38'04''E	5	4	0	0
SE	Haga-Haga	9	23°46'15''S, 28°14'16''E	2	2	1	1
SE	Morgan Bay	10	32°42'39''S 28°20'27''E	27	12	3	3
SE	Mngazana	11	31°41'41''S 29°25'27''E	1	6	0	3
E	Park Rynie	12	30°19'S 30°44'E	3	2	0	0
E	St Lucia	13	28°15'41''S, 32°29'47''E	6	12	3	3
Mozambique	Ponta do Ouro	14	26°50'40''S 32°53'43''E	2	4	0	0
Morocco	La Madrague	15	30°30'54''N, 9°44'48''W	14	6	0	0
	Immesouane	16	30°50'20''N, 9°49'23''W	18	6	0	0
Australia							
NSW	Fingal Head	17	28°11'56''S 153°34'16''E	20	38	1	1
	Ballina	18	28°52'05''S 153°35'36''E	19	0	0	0
	Port Macquarie	19	31°25'47''S 152°55'24''E	21	0	0	0
	Black Head	20	32°04'15''S 152°32'55''E	20	0	0	0
	Kiama	21	34°40'31''S 150°51'30''E	14	0	0	0
	Ulladulla	22	35°21'35''S 150°29'11''E	17	0	0	0
	Eden	23	37°04'01''S 149°54'47''E	16	0	0	0
VIC	Mallacoota	24	37°34'14''S 149°45'52''E	18	0	0	0
	Cape Conran	25	37°48'52''S 148°43'36''E	21	28	0	0
	Port Albert^8^	26	38°40'S 146°41'E	5	0	0	0
	Port Welshpool	27	38°42'04''S 146°27'54''E	8	20	1	1
	Walkerville^8^	28	38°51'49''S 146°00'08''E	5	0	0	0
	Kilcunda^8^	29	38°33'23''S 145°28'50''E	30	30	1	2
	Stoney Point^8^	30	38°22'21''S 145°13'30''E	4	0	0	0
	Hastings^8^	31	38°18'30''S 145°11'57''E	5	0	0	0
	Mornington^8^	32	38°12'49''S 145°02'04''E	3	0	0	0
	Portsea^8^	33	38°19'07''S 144°42'44''E	8	0	0	0
	Marengo Bay^8^	34	38°46'41''S 143°39'60''E	3	0	0	0
	Portarlington^8^	35	38°06'45''S 144°39'06''E	4	0	0	0
TAS	Beauty Point	36	41°09'S 146°49'E	8	22	3	3
	Two Tree Point	37	43°20'S 147°19'E	2	8	0	0
	Taroona Beach^9^	38	42°57'S 147°21'E	0	4	0	0
SA	Henley Beach	39	34°55'11''S 138°29'31''E	8	6	3	3
	Largs Bay^8^	40	34°47'48''S 138°29'04''E	4	0	0	0
	Brighton Beach^8^	41	35°01'03''S 138°30'46''E	4	0	0	0
WA	Albany	42	35°01'57''S 117°53'25''E	10	6	3	3
New Zealand	N Twilight Beach^9^	43	34°29'22''S 172°40'56''E	0	8	0	2
	S Twilight Beach^9^	44	34°30'32''S 172°41'59''E	0	6	0	1
	Tauroa Peninsula^9^	45	35°10'12''S 173°06'22''E	0	20	0	0
	N Herekino^9^	46	35°15'13''S 173°07'11''E	0	20	0	0
	The Bluff^9^	47	34°41'06''S 172°53'23''E	0	20	0	0
	Te Werahi Beach^9^	48	34°28'10''S 172°39'26''E	0	6	0	0
	Tarawamaomao Pt.^9^	49	34°26'12''S 172°40'30''E	0	4	0	0
Chile	Antofagasta	50	23°42'25''S 70°25'51''E	15^1^	52	3	3
Outgroup							
							
*Pyura dura*				1^2^	0	1	1^4^
*P. haustor*				0	0	0	1^5^
*P. spinifera*				1	1	1	1
*P. squamulosa*				1^3^	0	0	1^6^
							
			Total no. sequences:	403	411^7^	34	40

COI sequences were generated for most specimens. The ANT gene was primarily used to confirm genetic structure identified using COI and to study genetic diversity in selected populations. The less informative ATPSα and 18S were used for phylogeny reconstructions only in conjunction with COI and ANT. ^1^For calculations in Table 3, 6 COI sequences generated in Castilla et al. [34] were included; sequences downloaded from GenBank (not included in the total number of sequences generated): ^2^FJ528618, ^3^FJ528625, ^4^FM244856, ^5^AY90392, ^6^FM897341; ^7^ANT sequences were generated for 206 individuals. Both phases were resolved (except in the outgroup species *P. spinifera*), resulting in a total of 411 ANT sequences. ^8^Sites from which previously generated, unpublished COI sequences were incorporated into this study. ^9^Sites for whose samples no COI sequences could be generated, possibly due to a mutation in the primer annealing site. Acronyms: E = East, N = North, NSW = New South Wales, S = South, SA = South Australia, SE = south-east, SW = south-west, TAS = Tasmania, VIC = Victoria, W = West, WA = Western Australia.

We amplified one mitochondrial DNA (mtDNA) marker, the cytochrome oxidase subunit I gene (COI), and three nuclear DNA (nrDNA) markers: 18S (a component of the 40S cytoplasmic small ribosomal subunit in eukaryotes) and two nuclear genes containing introns, namely ATP synthase subunit α (ATPSα) and Adenine Nucleotide Transporter (ANT, also known as ADP/ATP translocase) (Table 2). We also used unpublished COI sequence data generated previously by some of our collaborators, and incorporated some published sequence data (Table 1). The COI gene was the primary marker used for identifying genetic lineages due to its high variability. ANT was the most variable nuclear marker. It was primarily used to confirm genetic structure identified using COI by amplifying a sub-set of samples, to study genetic diversity in selected populations, and to provide an alternative for COI in the few cases where this marker did not amplify due to a possible mutation in the primer annealing region (two samples from Tasmania and all samples from New Zealand, Table 1). ATPSα and 18S were less informative and for that reason were only used for phylogeny reconstructions.

**Table 2:** Genetic markers, primer sequences, and primer-specific annealing temperatures (T_a_) and MgCl_2 _concentrations

Marker	Primer names	Primer sequences (5'-3')	T_a_ (°C)	MgCl_2 _(mM)	References
COI	*LCO1490* *HCO2198*	TAAACTTCAGGGTGACCAAAAAATCA GGTCAACAAATCATAAAGATATTGG	50	6	[91] [91]
ANT	*StolidoANT-F* *ANTr1*	CAGGGTATCATTGTRTACMGAG CCAGACTGCATCATCATKCGRCGDC	60	3	This study [44]
ATPSα	*ATPSαf1* *ATPSαr1*	GAGCCMATGCAGACTGGTATTAAGGCYGT CTGTGGTAGTAGTTGGTCTTCKCNAAGTT	55	3	[44] [44]
18S	*5'F* *557F* *1262R* *3'R*	TYCCTGGTTGATYYTGCCAG GCCAGCAGCCGCGGT GGTGGTGCATGGCCGTY TGATCCATCTGCAGGTYCACCT	54	3	[92] [93] [93] [92]

Most of the primers used are universal. The ANT gene did not amplify readily using published primers [44], and a forward primer was designed to amplify it in Stolidobranchia ascidians (*StolidoANT-F*) in conjunction with a universal reverse primer (Table 2). This primer combination proved particularly useful for both phylogenetic and phylogeographic work, as the PCR product amplified reliably and contained a long, variable intron. The primer combination developed here amplified the ANT gene not only in *Pyura *spp., but also in other genera within the order Stolidobranchiata, including *Botrylloides *(GenBank accession number JF962229), *Botryllus *(JF962231) and *Styela *(JF962232).

PCR reaction conditions comprised 1 μl of template DNA (~150 ng), 3 μl of reaction buffer (Promega), 6 μl of dNTP mixture containing 125 mM of each dNTP, 1.2 μl of each primer (5 mM dilutions), 1 unit of Taq DNA polymerase (Promega, Madison, USA) and ddH_2_0 to a final volume of 30 μl. Concentrations of MgCl_2 _differed for each marker (Table 2). PCR profiles consisted of an initial denaturing step (94°C for 3 min), 35 cycles of denaturing (94°C for 30 s), annealing (45 s at a primer-specific annealing temperature, T_a_; Table 2) and extension (72°C for 45 s), and a final extension step (72°C for 10 min). The problem of PCR reactions being affected by low purity of DNA extractions could be circumvented by diluting DNA templates, which supposedly reduced potential inhibitors to a level at which they no longer interfered with the PCR reaction. Nonetheless, a quality screening procedure was applied in which samples were excluded when the quality of their trace files was too low to identify each nucleotide with absolute certainty in three successive sequencing runs. As ANT tended to amplify more reliably than COI, this explains why at some sites, more ANT sequences than COI sequences were generated, even though only a fraction of samples was sequenced using this marker (Table 1). PCR products were purified using the UltraClean^TM^ 15 DNA Purification Kit (MO BIO Laboratories, Inc., Carlsbad, CA, USA), sequenced in both directions using Big Dye terminator chemistry version 3.1 (Applied Biosystems, USA) and run on a 3130xl Genetic Analyser.

### Phase determination and alignment

In heterozygous individuals whose two ANT alleles differed in length, we used CHAMPURU v1.0 [45] to determine each phase. In cases where there were no length differences, each sequence was deduced using default settings for multi-allelic loci without stepwise mutation in PHASE v2.1 [46]. Whenever there were multiple possible phases, we selected the two alleles having the highest probability, which tended to be an order of magnitude greater than the probabilities of all other sequence pairs. Using alternative phases made no obvious difference in terms of estimates of genetic diversity and phylogenetic reconstructions.

COI and 18S sequences were aligned by eye in MEGA4 [47]. The nuclear genes containing introns were aligned using the multiple sequence alignment program T-COFFEE[48] at the BIOHPC website (http://cbsuapps.tc.cornell.edu). Poorly aligned regions were eliminated using GBLOCKS[49] by specifying the least stringent conditions at the GBLOCKS server (http://molevol.cmima.csic.es/castresana/GBLOCKS_server.html).

### Phylogeny reconstructions

Phylogenetic relationships among lineages of *Pyura stolonifera *were reconstructed using an aligned data-set from combined sequence data of four loci, totaling 2611 bp in length. After exploring the phylogenetic signal of data-sets from each locus, phylogenetic trees based on combined sequence data were reconstructed using no more than two representatives per lineage and region (14 ingroup taxa and four outgroup taxa). In several cases, regional lineages identified were excluded from the combined analyses because sequence data were not available for all four loci (Table 1). Three methods of phylogenetic reconstruction were employed and results compared: minimum evolution and parsimony, both employed in MEGA4, and Bayesian inference employed in MRBAYES 3.1 [50]. Support for nodes in the minimum evolution and parsimony trees was assessed by means of 10 000 bootstrap replications. In Bayesian inference, four chains of three million generations each were run simultaneously and trees were sampled every 100 generations. After removing the first 10% of trees as burn-in, posterior probabilities of nodes were assessed by constructing a 50% majority rule consensus tree. To check for consistency of results, the analyses were repeated three times. For Bayesian inference, the data-set was divided into 14 partitions: codon positions 1-3 of COI (534 bp), 18S (1703 bp), codon positions 1-3 for the ANT exon (81 bp) and the ATPSα exon (105 bp), ANT intron (137 bp), ATPSα intron (51 bp), ANT indels (19 characters) and ATPSα indels (8 characters). Rates were allowed to vary among nucleotide partitions, and the GTR+I+Γ model was specified for each. Information from indels was only incorporated when these that had clearly defined alignment boundaries.

In several cases, lower-level phylogenetic relationships were inferred using sequence data from single loci by either constructing neighbour-joining trees [51] in MEGA4 using maximum composite likelihoods [52] of Tamura-Nei distances [53] or by constructing median-joining haplotype networks in NETWORK4516 (2009 version) [54]. Indels were coded as single nucleotide differences irrespective of their length.

### Population comparisons

In populations that were not clearly differentiated on the basis of being comprised of monophyletic clades or clusters in haplotype networks, we estimated genetic diversity indices and calculated fixation indices to determine whether their allele frequencies differed. We used our two most variable markers, COI and ANT, to calculate the following statistics in ARLEQUIN v3.5 [55]: *h *(haplotype or gene diversity), π (nucleotide diversity), and pairwise fixation indices as a measure of population differentiation (Φ_ST _for COI and *F*_ST _for ANT). In addition, we estimated observed and expected heterozygosity of ANT sequences in each population.

We used molecular dating to determine whether divergence of closely related populations that are represented in different regions likely occurred during historical times (i.e. as a result of a human-mediated introduction) or whether they have more ancient origins (i.e. divergence > 2000 years ago). Divergence times among several pairs of populations were estimated under the isolation-with-migration model [56] using the program IMa [57]. We limited ourselves to populations that were either significantly differentiated on the basis of fixation indices, or that were comprised of reciprocally monophyletic sister clades. As two of the markers (ATPSα and 18S) showed little or no differentiation at the lowest taxonomic level, we used either a combination of COI and ANT, or COI only when ANT showed too little genetic variation to estimate divergence times. The population from New Zealand is represented by a single allele that was not found in its genetically most similar population in Australia (see Results) but is likely to be present there (see Discussion). For that reason, we did not estimate divergence times in this taxon. The HKY model [58] was selected as the best-fitting model for both markers by FINDMODEL[59], inheritance scalars of 0.25 for COI and 1 for ANT were specified, and a generation time of one year was assumed [60]. To our knowledge, there are as yet no published evolutionary rates for the COI gene of ascidians, but evolutionary rates between 0.5 and 1.5% per million years (Myr^-1^) are assumed in most studies on other marine invertebrate taxa [61,62]. To incorporate uncertainty, we specified a COI rate of 1% Myr^-1^ and incorporated a range from 0.5-1.5% Myr^-1^. The program does not require evolutionary rates to be specified for all markers included in an analysis. To check for the consistency of results, IMa runs were repeated three times using the following command line specifications: -l 25 000 (25 000 trees, with trees sampled every 100th generation, i.e. a total of 2.5 × 10^6^ generations) -b 100 000 (deletion of the first 100 000 generations as burn-in), -q1 500 (maximum θ = effective population size parameter scaled to evolutionary rate), -t 2 (maximum divergence time scaled to evolutionary rate), -m1 20 (migration into population 1) -m2 20 (migration into population 2), -f g -n 80 -g1 0.999 -g2 0.3 (geometric heating scheme with 80 heated chains and heating parameters of 0.999 and 0.3).

## Results

### Identification of evolutionary lineages

**Figure 1:**
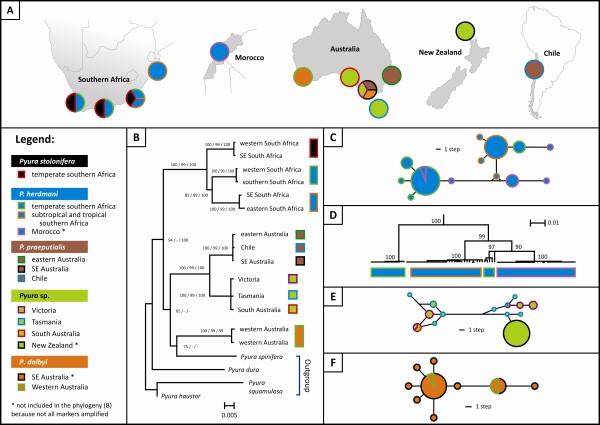
**Genetic lineages within the *Pyura stolonifera *species complex**. A) regions in which members of the species complex were collected for this study (see Table 1 for details); B) minimum evolution tree based on combined sequence data from 4 loci; support for nodes is indicated as bootstrap values (≥ 50%) from minimum evolution and parsimony analyses, and as posterior probabilities (≥ 95%) from Bayesian inference; C) haplotype network constructed from ANT sequences of *P. herdmani *and D) linearised neighbor-joining phylogeny based on sequences of the COI gene of *P. herdmani*; bootstrap values are indicated, and *P. stolonifera *was used as outgroup (not shown); E) haplotype network of ANT sequences of *Pyura *sp. and F) haplotype network of COI sequences of *P*. *dalbyi*. (Acronyms: ANT = nuclear Adenine Nucleotide Transporter gene; COI = mitochondrial cytochrome oxidase subunit I gene; SE = southeastern).

A total of 888 DNA sequences were generated, including 403 COI sequences, 411 ANT sequences, 34 ATPSα sequences and 40 18S sequences (Table 1). Most sequences (excluding some ANT sequences that were < 200 bp in length) were submitted to GenBank (accession numbers JF961754 - JF962415, see Additional File 1). Complete data-sets of aligned sequences are available in the following additional files: COI: Additional File 2; ANT: Additional File 3; ATPSα: Additional File 4 and 18S: Additional File 5. Additional File 6 contains combined sequence data used for phylogeny reconstruction. Six monophyletic clades were recovered with high support (bootstrap values and Bayesian posterior probabilities ≥ 99%, Figure 1b). Taxonomic descriptions exist for four of these clades (see Discussion for details). The African species *Pyura stolonifera *sensu stricto and *P. herdmani *formed a well-supported clade, and we also found strong support for the monophyly of two clades comprised of samples from Australasia and Chile (*P. praeputialis *and *Pyura *sp., Figure 1b). The placement of a third species present in Australasia (*P. dalbyi*, western and southeastern Australia) remains unresolved (Figure 1b). Support for its monophyly with the other two Australasian species was high in phylogenies of two of the more slowly-evolving partitions (e.g. ANT gene, Minimum Evolution Bootstrap support: 96%, 18S: 96%) and such a taxonomic placement is also supported by morphological data (see Discussion).

Regional sub-structuring was identified within *Pyura herdmani *and *Pyura *sp. on the basis of phylogenetic trees or haplotype networks constructed using sequence data from single markers. *Pyura herdmani *was comprised of four distinct lineages on the basis of mtDNA COI sequences (Figure 1d), although the more slowly-evolving nrDNA ANT sequences showed little differentiation among regions (Figure 1c), possibly due to incomplete lineage sorting. One of the lineages recovered using COI sequences is confined to Morocco, two occur in temperate South Africa, and the fourth is restricted to subtropical and tropical regions of southern Africa (south-eastern and eastern South Africa, and southern Mozambique). We included representatives of only two lineages in the phylogenetic tree based on combined sequence data (Figure 1b) to indicate that genetic differentiation among them is not much lower than among the taxa we considered to be distinct species, but it is important to note that there is presently not enough data to recognise any additional species within *P. herdmani*. The Australasian species *Pyura *sp. was comprised of two closely related ANT lineages of which one is found in Australia (Victoria, Tasmania and South Australia) and the other occurs both in Australia (Victoria and Tasmania) and in New Zealand (Figure 1e). Genetic diversity in Australia was high (13 unique alleles in 32 specimens), whereas all 42 individuals from New Zealand had the same ANT allele. We identified four heterozygous individuals in Australia having alleles from both lineages, suggesting that these are not distinct species.

### Population comparisons

Two pairs of geographically distant populations were not recovered as being distinct on the basis of phylogenetic trees or haplotype networks: Australian vs. Chilean representatives of *Pyura praeputialis *(Figure 1b) and Western Australian vs. southeastern Australian representatives of *P*. *dalbyi *(Figure 1f). Genetic diversity statistics were similar for all four populations of *P. praeputialis *investigated (three populations from Australia and one from Chile, Table 3). In most cases, genetic diversity estimates for the supposedly recently introduced population from Antofagasta, Chile, were the second highest of all the populations studied, and heterozygosity of this population at the diploid, intron-containing ANT gene was not lower than that of the Australian populations. On the basis of both pairwise Φ_ST _values among the mtDNA COI haplotypes and pairwise *F*_ST _values among the alleles of the nuclear ANT gene, we found significant structure between a site in southeastern Australia (Kilcunda) and all other sites, including the Chilean site Antofagasta. Significant structure between southeastern Australia and the two other regions, but not between the Australian east coast and Antofagasta, was also found when using a larger COI data-set that included samples from additional sites in eastern (sites 18 - 24 in Table 1) and southeastern (sites 28, 33 and 34) Australia for which no ANT data were generated (*N *= 232; southeast coast vs. east coast: Φ_ST _= 0.070, *P *< 0.01, southeast coast vs. Antofagasta: Φ_ST _= 0.070, *P *< 0.01; east coast vs. Antofagasta: Φ_ST _= -0.003, *P *= 0.51). This supports the idea that the significant genetic structure found between the two eastern Australian sites and the one southeastern Australian site is not an artifact of small samples sizes.

**Table 3:** Genetic diversity at four sites inhabited by *Pyura praeputialis*, and tests for genetic structure among them

Marker	Statistic		Sampling site			
			1 Fingal	2 Cape Conran	3 Kilcunda	4 Antofagasta

						
COI	*h*		0.968 ± 0.028	0.965 ± 0.024	0.835 ± 0.037	0.971 ± 0.024
	π		0.007 ± 0.004	0.006 ± 0.003	0.005 ± 0.003	0.007 ± 0.004
						
	Φ_ST_	1				
		2	0.001			
		3	0.060*	0.103**		
		4	-0.024	0.005	0.087**	
						
ANT	*h*		0.949 (± 0.017)	0.849 (± 0.033)	0.864 (± 0.041)	0.904 (± 0.023)
	π		0.014 (± 0.009)	0.009 (± 0.006)	0.011 (± 0.007)	0.013 (± 0.008)
	H_obs_		0.153 (± 0.141)	0.214 (± 0.151)	0.156 (± 0.151)	0.205 (± 0.146)
	H_exp_		0.197 (± 0.186)	0.305 (± 0.162)	0.161 (± 0.149)	0.245 (± 0.178)
						
	*F*_ST_	1				
		2	-0.002			
		3	0.085**	0.044*		
		4	-0.004	0.007	0.061*	
						

Site numbers refer to: 1 - Fingal (northern east coast of Australia); 2 - Cape Conran (southern east coast of Australia, east of the former Bassian Isthmus); 3 - Kilcunda (southeastern Australia, west of the former Bassian Isthmus); 4 - Antofagasta (Chile). Number of sequences generated for each site and genetic marker: Fingal: COI = 20, ANT = 38; Cape Conran: COI = 21, ANT = 28; Kilcunda: COI = 30, ANT = 30; Antofagasta: COI = 21 (including 6 sequences from Castilla et al. [34]), ANT = 52. Statistics include: *h *- haplotype or gene diversity; π - nucleotide diversity; Φ_ST _- pairwise fixation index among sites based on mtDNA COI haplotypes; *F*_ST _- pairwise fixation index among sites based on nrDNA ANT alleles; H_obs _and H_exp _- observed and expected mean heterozygosity based on ANT sequence data. Asterisks indicate significant fixation indices (*P < 0.05, **P < 0.01) and values in brackets are standard deviations.

No structure was identified between representatives of *P*. *dalbyi *from Western Australia vs. southeastern Australia (Φ_ST _= 0.06, *P *= 0.18), and diversity indices were similar (Western Australia: *h *= 1.000 ± 0.045, π = 0.005 ± 0.003, southeastern Australia: *h *= 1.000 ± 0.017, π = 0.004 ± 0.003; COI sequence data only).

A divergence time estimate of 1.1 million years ago (95% confidence interval: 0.4 - 2.4 million years ago) was estimated for the Moroccan population of *P. herdmani *and its temperate southern African sister lineage (Figure 1c). Although the eastern and southeastern Australian populations of *P. praeputialis *shared haplotypes, they were also estimated to have diverged prior to the historical period (150 thousand years ago with a 95% confidence interval of 79 - 420 thousand years).

## Discussion

In the present study, we show that the widespread ascidian *Pyura stolonifera *is a species complex that comprises at least five distinct species. Within some of these, we found additional genetic structure at regional scales, and we identified three populations that are likely to be non-indigenous. The species associated with the species complex are ecosystem engineers that create habitat complexity, compete with other sessile species for food and space and tend to be highly abundant once established [15,63]. As introduced ascidians can fundamentally alter both the structure and composition of benthic communities [15,64], reduce the diversity of native species [65] and threaten economically important species [39,66], it is imperative to define whether they are native or introduced in any particular region. However, phylogenetic and phylogeographic information may often be insufficient to determine this conclusively, even in conspicuous species such as the members of the *P. stolonifera *species complex.

### Species within *Pyura stolonifera*

In the taxonomic literature, three species associated with the *Pyura stolonifera *species complex have traditionally been considered, namely *Pyura stolonifera *sensu stricto, *P. herdmani *(Drasche, 1884) and *P. praeputialis *(Heller, 1987), but the validity of the latter two has been challenged [35,36] and their species names are not consistently applied [67]. For example, Castilla et al. [34] found genetic differentiation between the African *P. herdmani *(probably misidentified as *P. stolonifera *[see 67]), and populations from eastern Australia and Chile, and recommended referring to the latter as *P. praeputialis*. However, most subsequent studies continued to refer to the eastern Australian population as *P. stolonifera, *e.g. [68-77]. We found that *P. stolonifera *sensu stricto is restricted to temperate southern Africa (Figure 1) and that its range overlaps with that of *P. herdmani*, which occurs in temperate, subtropical and tropical southern Africa, as well as Morocco (Table 4). Confirming the findings of Castilla et al. [34], we found that *P. praeputialis *is both morphologically [67] and genetically (this study) distinct from its African congeners. The extensive sampling in Australasia revealed the existence of two more species within the species complex, namely *P. dalbyi *Rius & Teske, 2011 [67] and *Pyura *sp. (Victoria, South Australia, Tasmania and New Zealand), which has yet to be formally described.

**Table 4:** Regional genetic lineages identified in this study, names used in species description that match their morphology best, and assessment whether they are likely to be native or introduced in each region

Species/Lineage	Region	Sites^1^	Native/Introduced
			
*Pyura stolonifera*	South Africa		
	SW	2,3	Native
	S	4,6-8	Native
	SE	9	Native
			
*Pyura herdmani*	South Africa		
(Temperate)	SW	1,2	Native
	S	5,6,8	Native
	SE	10	Native
			
*Pyura herdmani*	South Africa		
(Subtropical/	SE	10,11	Native
Tropical)	E	12,13	Native
	Mozambique	14	Native
			
*Pyura herdmani*	Morocco	15,16	Native
(Moroccan)			
			
*Pyura praeputialis*	Australia		
	NSW	17-23	Native
	VIC	24,25,28,29,33,34	Native
	Chile	50	Introduced
			
*Pyura dalbyi*	Australia		
	VIC	27,30-32,35	Native
	WA	42	Introduced?
			
*Pyura *sp.	Australia		
	VIC	26,27	Native
	TAS	36-38	Native
	SA	39-41	Native
	New Zealand	34-49	Introduced

^1^Site numbers and acronyms correspond to those used in Table 1.

Three of the species identified (*Pyura herdmani, P. praeputialis *and *Pyura *sp.) can be further subdivided into regional genetic lineages on the basis of reciprocal monophyly or differences in allele frequencies, and the populations at several localities are likely to be the product of long-distance dispersal. These issues are discussed in the following two sections.

### Long-distance dispersal

Based on our genetic data, four populations may be the product of long-distance colonisation events, as they are genetically very similar to populations that are isolated from them by large geographic distances. These include the populations in Chile, New Zealand, Morocco and Western Australia. However, these populations differ considerably from each other both in terms of genetic diversity and in terms of how genetically distinct they are from their putative source populations.

Castilla et al. [34] found that the populations in eastern Australia and Antofagasta, Chile, are genetically very similar, but we considered this to be insufficient to conclude that the Chilean population has recently been introduced, as it could also indicate incomplete lineage sorting among significantly differentiated populations. It was thus considered necessary to obtain larger sample sizes from each population and to compare genetic diversity of the different populations. Lack of genetic structure between *P. praeputialis *from sites in eastern Australia and Chile supports the idea that the Chilean population is the product of a recent introduction, despite its high genetic diversity. Invasive marine invertebrates can show high genetic diversity due to multiple introductions of large numbers of individuals from different sources, e.g. [78,79] and the Chilean population includes a random sample of haplotypes found in eastern Australia. A very different result was found for *Pyura *sp., which has only recently been reported from New Zealand [39]. All 42 individuals from New Zealand had the same ANT allele, indicating loss of diversity through genetic drift or a strong bottleneck effect. The allele found in New Zealand is part of a cluster of haplotypes present in southeastern Australia, and the population in New Zealand clearly does not represent a distinct species. The Western Australian population of *P. dalbyi *was probably recently founded by individuals from southeastern Australia. Distribution records indicate that this species is absent from the Great Australian Bight [36] and thus has a disjunct distribution typical of an introduced species, with c 2500 km between its two regional populations. Even more compellingly, while *P. dalbyi *is common in Victoria (Table 1), it seems to be confined to only two Western Australian sites that are more than 1000 km apart [36]. At one of these (Albany), it has been found exclusively inside the harbour, suggesting that it has failed to spread beyond this point of introduction.

Thus we have at least three populations (in Chile, New Zealand and Western Australia, Table 4) that have apparently been recently introduced through human activities and that should be controlled if possible, even though they may provide biogenic habitat for other species.

Lastly, the Moroccan population of *P. herdmani *was recovered as a distinct lineage with high nodal support using the most quickly evolving marker used in this study, mtDNA COI. Molecular dating indicated that, like several other marine invertebrates with similar antitropical distributions [80,81], it diverged from its southern African sister lineage prior to the Holocene.

### Cryptic divergence within regions

In several cases, we identified genetic sub-structure within individual taxa (*Pyura herdmani*, *P. praeputialis *and *Pyura *sp.) that may point to the existence of additional cryptic species. While there is no evidence that any of these have become invasive elsewhere, their existence highlights the importance of sampling throughout the entire native range of a taxon suspected of having become invasive. Failure to capture all of the genetic diversity present within a particular region will result in a recently introduced species being mistaken for an indigenous species that was previously overlooked.

The most clear-cut example of cryptic divergence was found in *Pyura herdmani *between the temperate and subtropical/tropical provinces in southern Africa, which are inhabited by distinct lineages whose ranges overlap on the southeast coast. Phylogeographic disjunctions that coincide with water temperature have been documented in this region for various other marine organisms, and claims that the genetic lineages identified constitute cryptic species could in several cases be supported by morphological and physiological data [82-84]. In *Pyura *sp., two closely related lineages were identified. The fact that both were present at the same sites in Victoria and Tasmania and that several individuals had ANT alleles from both lineages, indicates that if regional genetic structure ever existed, it has almost completely eroded as a result of subsequent high levels of gene flow. Lastly, in *P. praeputialis *we found a very recently established genetic disjunction (based on allele frequency differences) across the Bass Strait. Phylogeographic breaks in this region have been documented for a large number of marine species [85,86] and in most cases, allopatric speciation due to the rise of the former Bassian Isthmus that connected Tasmania with the Australian mainland during periods of low sea-level has been invoked [85].

## Conclusion

Distinguishing a putative invader from a previously overlooked cryptic species can be a challenging task. Our results highlight the importance of extensive sampling to differentiate between native and introduced ranges in widespread marine invertebrates and illustrate the difficulty of correctly identifying non-indigenous species in marine invertebrates with poorly resolved taxonomy.

When attempting to match a population that is suspected of having been recently introduced to a source population, samples meant to represent a particular region usually originate from only a small portion of a species' local range, e.g. [28,34,87]. As there may be considerable variation in habitat quality along the range of widely distributed coastal species [88], such a sampling design can result in incorrect conclusions being drawn on whether populations are exotic or native when multiple genetic lineages are present within regions. In our case, some of the species identified have a preference for sheltered conditions (*Pyura herdmani*, *P. dalbyi *and *Pyura *sp.), whereas others can also be found at exposed sites on the open coast (*P. stolonifera *and *P. praeputialis*) [67]. Even more importantly, the fact that co-distributed coastal invertebrates in Australia, South Africa and North America tend to have congruent phylogeographic patterns that are often linked to well-documented marine biogeographic disjunctions, e.g. [84-86,89,90], indicates that it is crucial to collect samples in all biogeographic provinces in which a widespread species is represented (e.g. *P. herdmani*). To achieve good sampling cover, it is thus necessary to collect samples at as many sites as possible rather than obtaining large numbers of samples from a small number of sites. The latter approach is commonly used in population genetic studies in order to accurately estimate genetic diversity at each site, but such information is of little value when the aim of a study is to identify the source population of a putative invader.

Failure to identify and control a non-indigenous species could lead to habitat monopolisation at the expense of native species (e.g. in our study the populations in Chile, New Zealand and Western Australia), while the removal of an organism mistakenly identified as being invasive would constitute habitat destruction and may even result in the extinction of a native species (e.g. *Pyura herdmani *in Morocco). An inadequate sampling design, in which large numbers of sequences are generated but not all of the evolutionary lineages present in a particular region are recovered, can give researchers a false sense of confidence about the alien or indigenous status of poorly known marine organisms. This may obstruct management efforts aimed at controlling an introduced species during the critical early stages of an invasion.

## Authors' contributions

PRT designed the study, collected most of the Australian samples, generated most of the sequence data, did the analyses and prepared the manuscript. MR helped with the design of the study, provided most of the South African samples and helped with the laboratory work. CAS, MPP, SB, CFG, KW, MP, CFM, CRMA, GMC and SCB provided additional samples and generated additional sequence data. CMQ, NPB and LBB provided conceptual guidance and logistical support. All authors contributed to the preparation of the manuscript, and read and approved the final version.

## Supplementary Material

Additional file 1 GenBank accession numbers. Click here for file

Additional file 2 COI sequences. Click here for file

Additional file 3 ANT sequences. Click here for file

Additional file 4 ATPSα sequences. Click here for file

Additional file 5 18S sequences. Click here for file

Additional file 6 Combined sequence data used for phylogeny reconstructions. Click here for file
