# Supplementary material for: "Nested" cryptic diversity in a widespread marine ecosystem engineer: a challenge for detecting biological invasions
Source: BMC Evol Biol. 2011 Jun 21;11:176. doi: 10.1186/1471-2148-11-176 (PMC3146881; doi:10.1186/1471-2148-11-176)
Supplement: Additional file 1 — GenBank accession numbers. [file 1471-2148-11-176-S1.DOC]

| **Additional File 1: GenBank accession numbers** | | |  |  |  |  |  |  |  |
| --- | --- | --- | --- | --- | --- | --- | --- | --- | --- |
| Species | Region | Subregion | Site No. | Sample Name | COI | ANT-phase1 | ANT-phase2 | ATPSa | 18S |
| Pyura stolonifera | South Africa | South-west | 2 | PstolYzerfontein1 | JF961827 |  |  |  |  |
|  |  |  | 2 | PstolYzerfontein3 | JF961828 |  |  |  |  |
|  |  |  | 2 | PstolYzerfontein4 | JF961829 |  |  |  |  |
|  |  |  | 2 | PstolYzerfontein5 | JF961830 |  |  |  |  |
|  |  |  | 2 | PstolYzerfontein6 | JF961831 |  |  |  |  |
|  |  |  | 2 | PstolYzerfontein7 | JF961832 |  |  |  |  |
|  |  |  | 2 | PstolYzerfontein30 |  | JF962233 | JF962234 | JF961754 | JF961787 |
|  |  |  | 2 | PstolYzerfontein31 | JF961833 | JF962235 | JF962236 | JF961755 | JF961788 |
|  |  |  | 2 | PstolYzerfontein32 | JF961834 | JF962237 | JF962238 |  |  |
|  |  |  | 2 | PstolYzerfontein34 | JF961835 |  |  |  |  |
|  |  |  | 2 | PstolYzerfontein36 | JF961836 |  |  |  |  |
|  |  |  | 2 | PstolYzerfontein50 |  | JF962239 | JF962240 |  |  |
|  |  |  | 2 | PstolYzerfontein51 |  | JF962241 | JF962242 |  |  |
|  |  |  | 2 | PstolYzerfontein52 |  | JF962243 | JF962244 |  |  |
|  |  |  | 2 | PstolYzerfontein53 |  | JF962245 | JF962246 |  |  |
|  |  |  | 2 | PstolYzerfontein56 |  | JF962247 | JF962248 |  |  |
|  |  |  | 2 | PstolYzerfontein57 |  | JF962249 | JF962250 |  |  |
|  |  |  | 2 | PstolYzerfontein58 |  | JF962251 | JF962252 |  |  |
|  |  |  | 2 | PstolYzerfontein59 |  | JF962253 | JF962254 |  |  |
|  |  |  | 3 | PstolFalseBay6 | JF961837 |  |  | JF961756 | JF961789 |
|  |  |  | 3 | PstolFalseBay31 |  | JF962255 | JF962256 |  |  |
|  |  |  | 3 | PstolFalseBay32 |  | JF962257 | JF962258 |  |  |
|  |  |  | 3 | PstolFalseBay35 |  | JF962259 | JF962260 |  |  |
|  |  | South | 4 | PstolMosselBay11 | JF961838 |  |  | JF961757 | JF961790 |
|  |  |  | 6 | PstolPlettenbergBay2 | JF961839 |  |  |  |  |
|  |  |  | 6 | PstolPlettenbergBay3 | JF961840 |  |  |  |  |
|  |  |  | 6 | PstolPlettenbergBay7 | JF961841 |  |  |  |  |
|  |  |  | 8 | PstolPortElizabeth1 | JF961842 |  |  |  |  |
|  |  |  | 7 | PstolTsitsikamma1 | JF961843 |  |  |  |  |
|  |  |  | 7 | PstolTsitsikamma6 | JF961844 |  |  |  |  |
|  |  | South-east | 9 | PstolHagaHaga1 | JF961845 | JF962261 | JF962262 | JF961758 | JF961791 |
|  |  |  | 9 | PstolHagaHaga2 | JF961846 |  |  |  |  |
| Pyura herdmani | South Africa | South-west | 1 | PherdLangebaan1 | JF961847 | JF962263 | JF962264 | JF961759 | JF961792 |
|  |  |  | 1 | PherdLangebaan2 | JF961848 |  |  |  |  |
|  |  |  | 1 | PherdLangebaan4 | JF961849 | JF962265 | JF962266 |  |  |
|  |  |  | 1 | PherdLangebaan21 | JF961850 | JF962267 | JF962268 | JF961760 | JF961793 |
|  |  |  | 1 | PherdLangebaan22 | JF961851 |  |  |  |  |
|  |  |  | 1 | PherdLangebaan23 | JF961852 |  |  |  |  |
|  |  |  | 1 | PherdLangebaan24 | JF961853 | JF962269 | JF962270 | JF961761 | JF961794 |
|  |  |  | 1 | PherdLangebaan25 |  | JF962271 | JF962272 |  |  |
|  |  |  | 1 | PherdLangebaan26 |  | JF962273 | JF962274 |  |  |
|  |  |  | 1 | PherdLangebaan27 |  | JF962275 | JF962276 |  |  |
|  |  |  | 1 | PherdLangebaan28 |  | JF962277 | JF962278 |  |  |
|  |  |  | 2 | PherdYzerfontein2 | JF961854 |  |  |  |  |
|  |  |  | 2 | PherdYzerfontein40 | JF961855 |  |  |  |  |
|  |  |  | 2 | PherdYzerfontein41 |  | JF962279 | JF962280 |  |  |
|  |  |  | 2 | PherdYzerfontein42 | JF961856 | JF962281 | JF962282 |  |  |
|  |  | South | 5 | PherdKnysna1 | JF961857 | JF962283 | JF962284 | JF961762 | JF961795 |
|  |  |  | 5 | PherdKnysna2 | JF961858 | JF962285 | JF962286 | JF961763 | JF961796 |
|  |  |  | 5 | PherdKnysna3 | JF961859 | JF962287 | JF962288 |  |  |
|  |  |  | 5 | PherdKnysna4 | JF961860 | JF962289 | JF962290 | JF961764 | JF961797 |
|  |  |  | 5 | PherdKnysna5 |  |  |  |  |  |
|  |  |  | 5 | PherdKnysna11 |  | JF962291 | JF962292 |  |  |
|  |  |  | 6 | PherdPlettenbergBay1 | JF961861 |  |  |  |  |
|  |  |  | 8 | PherdPortElizabeth1 | JF961862 | JF962293 | JF962294 |  |  |
|  |  |  | 8 | PherdPortElizabeth2 | JF961863 |  |  |  |  |
|  |  |  | 8 | PherdPortElizabeth3 | JF961864 | JF962295 | JF962296 |  |  |
|  |  |  | 8 | PherdPortElizabeth4 | JF961865 |  |  |  |  |
|  |  | South-east | 10 | PherdMorganBay1 | JF961866 |  |  |  |  |
|  |  |  | 10 | PherdMorganBay2 | JF961867 |  |  |  |  |
|  |  |  | 10 | PherdMorganBay3 | JF961868 |  |  |  |  |
|  |  |  | 10 | PherdMorganBay4 | JF961869 |  |  |  |  |
|  |  |  | 10 | PherdMorganBay6 | JF961870 |  |  |  |  |
|  |  |  | 10 | PherdMorganBay7 | JF961871 |  |  |  |  |
|  |  |  | 10 | PherdMorganBay8 | JF961872 |  |  |  |  |
|  |  |  | 10 | PherdMorganBay9 | JF961873 |  |  |  |  |
|  |  |  | 10 | PherdMorganBay10 | JF961874 |  |  |  |  |
|  |  |  | 10 | PherdMorganBay12 | JF961875 |  |  |  |  |
|  |  |  | 10 | PherdMorganBay13 | JF961876 |  |  |  |  |
|  |  |  | 10 | PherdMorganBay16 | JF961877 |  |  |  |  |
|  |  |  | 10 | PherdMorganBay17 | JF961878 |  |  |  |  |
|  |  |  | 10 | PherdMorganBay18 | JF961879 |  |  |  |  |
|  |  |  | 10 | PherdMorganBay19 | JF961880 |  |  |  |  |
|  |  |  | 10 | PherdMorganBay20 | JF961881 | JF962297 | JF962298 | JF961765 | JF961798 |
|  |  |  | 10 | PherdMorganBay21 | JF961882 | JF962299 | JF962300 | JF961766 | JF961799 |
|  |  |  | 10 | PherdMorganBay22 | JF961883 | JF962301 | JF962302 | JF961767 | JF961800 |
|  |  |  | 10 | PherdMorganBay23 | JF961884 | JF962303 | JF962304 |  |  |
|  |  |  | 10 | PherdMorganBay24 | JF961885 | JF962305 | JF962306 |  |  |
|  |  |  | 10 | PherdMorganBay25 | JF961886 |  |  |  |  |
|  |  |  | 10 | PherdMorganBay26 | JF961887 |  |  |  |  |
|  |  |  | 10 | PherdMorganBay27 | JF961888 |  |  |  |  |
|  |  |  | 10 | PherdMorganBay28 | JF961889 |  |  |  |  |
|  |  |  | 10 | PherdMorganBay29 | JF961890 | JF962307 | JF962308 |  |  |
|  |  |  | 10 | PherdMorganBay30 | JF961891 |  |  |  |  |
|  |  |  | 10 | PherdMorganBay31 | JF961892 |  |  |  |  |
|  |  |  | 11 | PherdMngazana5 |  | JF962309 | JF962310 |  | JF961801 |
|  |  |  | 11 | PherdMngazana7 |  | JF962311 | JF962312 |  | JF961802 |
|  |  |  | 11 | PherdMngazana10 | JF961893 | JF962313 | JF962314 |  | JF961803 |
|  |  | East | 12 | PherdParkRynie1 | JF961894 | JF962315 | JF962316 |  |  |
|  |  |  | 12 | PherdParkRynie2 | JF961895 |  |  |  |  |
|  |  |  | 12 | PherdParkRynie3 | JF961896 |  |  |  |  |
|  |  |  | 13 | PherdStLucia1 | JF961897 | JF962317 | JF962318 | JF961768 | JF961804 |
|  |  |  | 13 | PherdStLucia2 | JF961898 | JF962319 | JF962320 | JF961769 | JF961805 |
|  |  |  | 13 | PherdStLucia3 | JF961899 | JF962321 | JF962322 | JF961770 | JF961806 |
|  |  |  | 13 | PherdStLucia4 | JF961900 |  |  |  |  |
|  |  |  | 13 | PherdStLucia5 | JF961901 |  |  |  |  |
|  |  |  | 13 | PherdStLucia6 | JF961902 |  |  |  |  |
|  |  |  | 13 | PherdStLucia8 |  | JF962323 | JF962324 |  |  |
|  |  |  | 13 | PherdStLucia9 |  | JF962325 | JF962326 |  |  |
|  |  |  | 13 | PherdStLucia10 |  | JF962327 | JF962328 |  |  |
|  | Mozambique |  | 14 | PherdPontaDeOuro1 | JF961903 | JF962329 | JF962330 |  |  |
|  |  |  | 14 | PherdPontaDeOuro2 | JF961904 | JF962331 | JF962332 |  |  |
|  | Morocco |  | 15 | PherdLaMadrague1 | JF961905 |  |  |  |  |
|  |  |  | 15 | PherdLaMadrage3 |  | JF962335 | JF962336 |  |  |
|  |  |  | 15 | PherdLaMadrague4 | JF961906 |  |  |  |  |
|  |  |  | 15 | PherdLaMadrague5 | JF961907 |  |  |  |  |
|  |  |  | 15 | PherdLaMadrague6 | JF961908 |  |  |  |  |
|  |  |  | 15 | PherdLaMadrague7 | JF961909 |  |  |  |  |
|  |  |  | 15 | PherdLaMadrague8 | JF961910 |  |  |  |  |
|  |  |  | 15 | PherdLaMadrague9 | JF961911 |  |  |  |  |
|  |  |  | 15 | PherdLaMadrague10 | JF961912 |  |  |  |  |
|  |  |  | 15 | PherdLaMadrague11 | JF961913 |  |  |  |  |
|  |  |  | 15 | PherdLaMadrague12 | JF961914 |  |  |  |  |
|  |  |  | 15 | PherdLaMadrague13 | JF961915 |  |  |  |  |
|  |  |  | 15 | PherdLaMadrague14 | JF961916 |  |  |  |  |
|  |  |  | 15 | PherdLaMadrague16 | JF961917 |  |  |  |  |
|  |  |  | 15 | PherdLaMadrague18 | JF961918 |  |  |  |  |
|  |  |  | 15 | PherdLaMadrague21 |  | JF962337 | JF962338 |  |  |
|  |  |  | 15 | PherdLaMadrague22 |  | JF962333 | JF962334 |  |  |
|  |  |  | 16 | PherdImmesouane1 | JF961919 |  |  |  |  |
|  |  |  | 16 | PherdImmesouane3 | JF961920 |  |  |  |  |
|  |  |  | 16 | PherdImmesouane4 | JF961921 |  |  |  |  |
|  |  |  | 16 | PherdImmesouane5 | JF961922 |  |  |  |  |
|  |  |  | 16 | PherdImmesouane6 | JF961923 |  |  |  |  |
|  |  |  | 16 | PherdImmesouane7 | JF961924 |  |  |  |  |
|  |  |  | 16 | PherdImmesouane8 | JF961925 |  |  |  |  |
|  |  |  | 16 | PherdImmesouane9 | JF961926 |  |  |  |  |
|  |  |  | 16 | PherdImmesouane10 | JF961927 |  |  |  |  |
|  |  |  | 16 | PherdImmesouane11 | JF961928 | JF962339 | JF962340 |  |  |
|  |  |  | 16 | PherdImmesouane12 | JF961929 |  |  |  |  |
|  |  |  | 16 | PherdImmesouane13 | JF961930 |  |  |  |  |
|  |  |  | 16 | PherdImmesouane14 | JF961931 |  |  |  |  |
|  |  |  | 16 | PherdImmesouane15 | JF961932 |  |  |  |  |
|  |  |  | 16 | PherdImmesouane16 | JF961933 | JF962341 | JF962342 |  |  |
|  |  |  | 16 | PherdImmesouane17 | JF961934 | JF962343 | JF962344 |  |  |
|  |  |  | 16 | PherdImmesouane18 | JF961935 |  |  |  |  |
|  |  |  | 16 | PherdImmesouane19 | JF961936 |  |  |  |  |
| Pyura praeputialis | Australia | New South Wales | 17 | PpraeFingalHead1 | JF961944 |  |  |  |  |
|  |  |  | 17 | PpraeFingalHead2 |  |  |  |  |  |
|  |  |  | 17 | PpraeFingalHead4 | JF961937 |  |  |  |  |
|  |  |  | 17 | PpraeFingalHead5 | JF961938 |  |  |  |  |
|  |  |  | 17 | PpraeFingalHead6 | JF961939 |  |  |  |  |
|  |  |  | 17 | PpraeFingalHead7 | JF961940 |  |  |  |  |
|  |  |  | 17 | PpraeFingalHead8 | JF961941 |  |  |  |  |
|  |  |  | 17 | PpraeFingalHead9 | JF961942 | JF962345 |  | JF961771 | JF961809 |
|  |  |  | 17 | PpraeFingalHead10 |  |  |  |  |  |
|  |  |  | 17 | PpraeFingalHead11 | JF961943 |  |  |  |  |
|  |  |  | 17 | PpraeFingalHead12 | JF961945 |  |  |  |  |
|  |  |  | 17 | PpraeFingalHead13 | JF961946 |  |  |  |  |
|  |  |  | 17 | PpraeFingalHead14 | JF961947 |  |  |  |  |
|  |  |  | 17 | PpraeFingalHead15 | JF961948 |  |  |  |  |
|  |  |  | 17 | PpraeFingalHead16 |  |  |  |  |  |
|  |  |  | 17 | PpraeFingalHead17 | JF961949 |  |  |  |  |
|  |  |  | 17 | PpraeFingalHead18 | JF961950 |  |  |  |  |
|  |  |  | 17 | PpraeFingalHead19 | JF961951 |  |  |  |  |
|  |  |  | 17 | PpraeFingalHead20 | JF961952 |  |  |  |  |
|  |  |  | 17 | PpraeFingalHead21 | JF961953 |  |  |  |  |
|  |  |  | 17 | PpraeFingalHead22 | JF961954 |  |  |  |  |
|  |  |  | 17 | PpraeFingalHead23 | JF961955 |  |  |  |  |
|  |  |  | 17 | PpraeFingalHead24 | JF961956 |  |  |  |  |
|  |  |  | 18 | PpraeBallina1 | JF961957 |  |  |  |  |
|  |  |  | 18 | PpraeBallina2 | JF961958 |  |  |  |  |
|  |  |  | 18 | PpraeBallina3 | JF961959 |  |  |  |  |
|  |  |  | 18 | PpraeBallina4 | JF961960 |  |  |  |  |
|  |  |  | 18 | PpraeBallina5 | JF961961 |  |  |  |  |
|  |  |  | 18 | PpraeBallina6 | JF961962 |  |  |  |  |
|  |  |  | 18 | PpraeBallina7 | JF961963 |  |  |  |  |
|  |  |  | 18 | PpraeBallina8 | JF961964 |  |  |  |  |
|  |  |  | 18 | PpraeBallina9 | JF961965 |  |  |  |  |
|  |  |  | 18 | PpraeBallina10 | JF961966 |  |  |  |  |
|  |  |  | 18 | PpraeBallina11 | JF961967 |  |  |  |  |
|  |  |  | 18 | PpraeBallina13 | JF961968 |  |  |  |  |
|  |  |  | 18 | PpraeBallina14 | JF961969 |  |  |  |  |
|  |  |  | 18 | PpraeBallina16 | JF961970 |  |  |  |  |
|  |  |  | 18 | PpraeBallina17 | JF961971 |  |  |  |  |
|  |  |  | 18 | PpraeBallina19 | JF961972 |  |  |  |  |
|  |  |  | 18 | PpraeBallina21 | JF961973 |  |  |  |  |
|  |  |  | 18 | PpraeBallina23 | JF961974 |  |  |  |  |
|  |  |  | 18 | PpraeBallina24 | JF961975 |  |  |  |  |
|  |  |  | 19 | PpraePortMacquarie1 | JF961976 |  |  |  |  |
|  |  |  | 19 | PpraePortMacquarie2 | JF961977 |  |  |  |  |
|  |  |  | 19 | PpraePortMacquarie3 | JF961978 |  |  |  |  |
|  |  |  | 19 | PpraePortMacquarie4 | JF961979 |  |  |  |  |
|  |  |  | 19 | PpraePortMacquarie5 | JF961980 |  |  |  |  |
|  |  |  | 19 | PpraePortMacquarie8 | JF961981 |  |  |  |  |
|  |  |  | 19 | PpraePortMacquarie9 | JF961982 |  |  |  |  |
|  |  |  | 19 | PpraePortMacquarie10 | JF961983 |  |  |  |  |
|  |  |  | 19 | PpraePortMacquarie11 | JF961984 |  |  |  |  |
|  |  |  | 19 | PpraePortMacquarie12 | JF961985 |  |  |  |  |
|  |  |  | 19 | PpraePortMacquarie13 | JF961986 |  |  |  |  |
|  |  |  | 19 | PpraePortMacquarie14 | JF961987 |  |  |  |  |
|  |  |  | 19 | PpraePortMacquarie15 | JF961988 |  |  |  |  |
|  |  |  | 19 | PpraePortMacquarie16 | JF961989 |  |  |  |  |
|  |  |  | 19 | PpraePortMacquarie17 | JF961990 |  |  |  |  |
|  |  |  | 19 | PpraePortMacquarie19 | JF961991 |  |  |  |  |
|  |  |  | 19 | PpraePortMacquarie20 | JF961992 |  |  |  |  |
|  |  |  | 19 | PpraePortMacquarie21 | JF961993 |  |  |  |  |
|  |  |  | 19 | PpraePortMacquarie22 | JF961994 |  |  |  |  |
|  |  |  | 19 | PpraePortMacquarie23 | JF961995 |  |  |  |  |
|  |  |  | 19 | PpraePortMacquarie24 | JF961996 |  |  |  |  |
|  |  |  | 20 | PpraeBlackHead1 | JF961997 |  |  |  |  |
|  |  |  | 20 | PpraeBlackHead2 | JF961998 |  |  |  |  |
|  |  |  | 20 | PpraeBlackHead3 | JF961999 |  |  |  |  |
|  |  |  | 20 | PpraeBlackHead4 | JF962000 |  |  |  |  |
|  |  |  | 20 | PpraeBlackHead5 | JF962001 |  |  |  |  |
|  |  |  | 20 | PpraeBlackHead6 | JF962002 |  |  |  |  |
|  |  |  | 20 | PpraeBlackHead7 | JF962003 |  |  |  |  |
|  |  |  | 20 | PpraeBlackHead9 | JF962004 |  |  |  |  |
|  |  |  | 20 | PpraeBlackHead10 | JF962005 |  |  |  |  |
|  |  |  | 20 | PpraeBlackHead11 | JF962006 |  |  |  |  |
|  |  |  | 20 | PpraeBlackHead12 | JF962007 |  |  |  |  |
|  |  |  | 20 | PpraeBlackHead13 | JF962008 |  |  |  |  |
|  |  |  | 20 | PpraeBlackHead14 | JF962009 |  |  |  |  |
|  |  |  | 20 | PpraeBlackHead15 | JF962010 |  |  |  |  |
|  |  |  | 20 | PpraeBlackHead17 | JF962011 |  |  |  |  |
|  |  |  | 20 | PpraeBlackHead18 | JF962012 |  |  |  |  |
|  |  |  | 20 | PpraeBlackHead19 | JF962013 |  |  |  |  |
|  |  |  | 20 | PpraeBlackHead22 | JF962014 |  |  |  |  |
|  |  |  | 20 | PpraeBlackHead23 | JF962015 |  |  |  |  |
|  |  |  | 20 | PpraeBlackHead24 | JF962016 |  |  |  |  |
|  |  |  | 21 | PpraeKiama1 | JF962017 |  |  |  |  |
|  |  |  | 21 | PpraeKiama2 | JF962018 |  |  |  |  |
|  |  |  | 21 | PpraeKiama4 | JF962019 |  |  |  |  |
|  |  |  | 21 | PpraeKiama5 | JF962020 |  |  |  |  |
|  |  |  | 21 | PpraeKiama6 | JF962021 |  |  |  |  |
|  |  |  | 21 | PpraeKiama7 | JF962022 |  |  |  |  |
|  |  |  | 21 | PpraeKiama8 | JF962023 |  |  |  |  |
|  |  |  | 21 | PpraeKiama10 | JF962024 |  |  |  |  |
|  |  |  | 21 | PpraeKiama11 | JF962025 |  |  |  |  |
|  |  |  | 21 | PpraeKiama12 | JF962026 |  |  |  |  |
|  |  |  | 21 | PpraeKiama13 | JF962027 |  |  |  |  |
|  |  |  | 21 | PpraeKiama14 | JF962028 |  |  |  |  |
|  |  |  | 21 | PpraeKiama15 | JF962029 |  |  |  |  |
|  |  |  | 21 | PpraeKiama16 | JF962030 |  |  |  |  |
|  |  |  | 22 | PpraeUlladulla1 | JF962031 |  |  |  |  |
|  |  |  | 22 | PpraeUlladulla3 | JF962032 |  |  |  |  |
|  |  |  | 22 | PpraeUlladulla4 | JF962033 |  |  |  |  |
|  |  |  | 22 | PpraeUlladulla5 | JF962034 |  |  |  |  |
|  |  |  | 22 | PpraeUlladulla6 | JF962035 |  |  |  |  |
|  |  |  | 22 | PpraeUlladulla7 | JF962036 |  |  |  |  |
|  |  |  | 22 | PpraeUlladulla8 | JF962037 |  |  |  |  |
|  |  |  | 22 | PpraeUlladulla9 | JF962038 |  |  |  |  |
|  |  |  | 22 | PpraeUlladulla10 | JF962039 |  |  |  |  |
|  |  |  | 22 | PpraeUlladulla12 | JF962040 |  |  |  |  |
|  |  |  | 22 | PpraeUlladulla13 | JF962041 |  |  |  |  |
|  |  |  | 22 | PpraeUlladulla14 | JF962042 |  |  |  |  |
|  |  |  | 22 | PpraeUlladulla15 | JF962043 |  |  |  |  |
|  |  |  | 22 | PpraeUlladulla16 | JF962044 |  |  |  |  |
|  |  |  | 22 | PpraeUlladulla17 | JF962045 |  |  |  |  |
|  |  |  | 22 | PpraeUlladulla18 | JF962046 |  |  |  |  |
|  |  |  | 22 | PpraeUlladulla19 | JF962047 |  |  |  |  |
|  |  |  | 23 | PpraeEden2 | JF962048 |  |  |  |  |
|  |  |  | 23 | PpraeEden3 | JF962049 |  |  |  |  |
|  |  |  | 23 | PpraeEden5 | JF962050 |  |  |  |  |
|  |  |  | 23 | PpraeEden6 | JF962051 |  |  |  |  |
|  |  |  | 23 | PpraeEden7 | JF962052 |  |  |  |  |
|  |  |  | 23 | PpraeEden8 | JF962053 |  |  |  |  |
|  |  |  | 23 | PpraeEden11 | JF962054 |  |  |  |  |
|  |  |  | 23 | PpraeEden12 | JF962055 |  |  |  |  |
|  |  |  | 23 | PpraeEden13 | JF962056 |  |  |  |  |
|  |  |  | 23 | PpraeEden14 | JF962057 |  |  |  |  |
|  |  |  | 23 | PpraeEden15 | JF962058 |  |  |  |  |
|  |  |  | 23 | PpraeEden16 | JF962059 |  |  |  |  |
|  |  |  | 23 | PpraeEden18 | JF962060 |  |  |  |  |
|  |  |  | 23 | PpraeEden20 | JF962061 |  |  |  |  |
|  |  |  | 23 | PpraeEden21 | JF962062 |  |  |  |  |
|  |  |  | 23 | PpraeEden22 | JF962063 |  |  |  |  |
|  |  | Victoria | 24 | PpraeMallacoota1 | JF962064 |  |  |  |  |
|  |  |  | 24 | PpraeMallacoota2 | JF962065 |  |  |  |  |
|  |  |  | 24 | PpraeMallacoota3 | JF962066 |  |  |  |  |
|  |  |  | 24 | PpraeMallacoota4 | JF962067 |  |  |  |  |
|  |  |  | 24 | PpraeMallacoota5 | JF962068 |  |  |  |  |
|  |  |  | 24 | PpraeMallacoota6 | JF962069 |  |  |  |  |
|  |  |  | 24 | PpraeMallacoota7 | JF962070 |  |  |  |  |
|  |  |  | 24 | PpraeMallacoota8 | JF962071 |  |  |  |  |
|  |  |  | 24 | PpraeMallacoota9 | JF962072 |  |  |  |  |
|  |  |  | 24 | PpraeMallacoota10 | JF962073 |  |  |  |  |
|  |  |  | 24 | PpraeMallacoota12 | JF962074 |  |  |  |  |
|  |  |  | 24 | PpraeMallacoota13 | JF962075 |  |  |  |  |
|  |  |  | 24 | PpraeMallacoota14 | JF962076 |  |  |  |  |
|  |  |  | 24 | PpraeMallacoota15 | JF962077 |  |  |  |  |
|  |  |  | 24 | PpraeMallacoota17 | JF962078 |  |  |  |  |
|  |  |  | 24 | PpraeMallacoota21 | JF962079 |  |  |  |  |
|  |  |  | 24 | PpraeMallacoota22 | JF962080 |  |  |  |  |
|  |  |  | 24 | PpraeMallacoota23 | JF962081 |  |  |  |  |
|  |  |  | 25 | PpraeCapeConran1 | JF962082 |  |  |  |  |
|  |  |  | 25 | PpraeCapeConran2 |  |  |  |  |  |
|  |  |  | 25 | PpraeCapeConran3 |  |  |  |  |  |
|  |  |  | 25 | PpraeCapeConran4 | JF962083 |  |  |  |  |
|  |  |  | 25 | PpraeCapeConran5 | JF962084 |  |  |  |  |
|  |  |  | 25 | PpraeCapeConran6 |  |  |  |  |  |
|  |  |  | 25 | PpraeCapeConran7 | JF962085 |  |  |  |  |
|  |  |  | 25 | PpraeCapeConran8 |  |  |  |  |  |
|  |  |  | 25 | PpraeCapeConran9 | JF962086 |  |  |  |  |
|  |  |  | 25 | PpraeCapeConran10 | JF962087 |  |  |  |  |
|  |  |  | 25 | PpraeCapeConran11 | JF962088 |  |  |  |  |
|  |  |  | 25 | PpraeCapeConran12 | JF962089 |  |  |  |  |
|  |  |  | 25 | PpraeCapeConran14 |  |  |  |  |  |
|  |  |  | 25 | PpraeCapeConran15 |  |  |  |  |  |
|  |  |  | 25 | PpraeCapeConran16 | JF962090 |  |  |  |  |
|  |  |  | 25 | PpraeCapeConran17 | JF962091 |  |  |  |  |
|  |  |  | 25 | PpraeCapeConran22 | JF962092 |  |  |  |  |
|  |  |  | 25 | PpraeCapeConran23 | JF962093 |  |  |  |  |
|  |  |  | 25 | PpraeCapeConran24 | JF962094 |  |  |  |  |
|  |  |  | 25 | PpraeCapeConran25 | JF962095 |  |  |  |  |
|  |  |  | 25 | PpraeCapeConran26 | JF962096 |  |  |  |  |
|  |  |  | 25 | PpraeCapeConran27 | JF962097 |  |  |  |  |
|  |  |  | 25 | PpraeCapeConran28 | JF962098 |  |  |  |  |
|  |  |  | 25 | PpraeCapeConran29 | JF962099 |  |  |  |  |
|  |  |  | 25 | PpraeCapeConran30 | JF962100 |  |  |  |  |
|  |  |  | 25 | PpraeCapeConran31 | JF962101 |  |  |  |  |
|  |  |  | 25 | PpraeCapeConran32 | JF962102 |  |  |  |  |
|  |  |  | 29 | PpraeKilcunda1 | JF962108 |  |  |  | JF961807 |
|  |  |  | 29 | PpraeKilcunda2 | JF962109 |  |  |  |  |
|  |  |  | 29 | PpraeKilcunda3 | JF962110 |  |  |  |  |
|  |  |  | 29 | PpraeKilcunda4 | JF962111 |  |  |  |  |
|  |  |  | 29 | PpraeKilcunda5 |  |  |  |  |  |
|  |  |  | 29 | PpraeKilcunda6 | JF962112 |  |  |  |  |
|  |  |  | 29 | PpraeKilcunda7 | JF962113 |  |  |  |  |
|  |  |  | 29 | PpraeKilcunda8 | JF962114 |  |  |  |  |
|  |  |  | 29 | PpraeKilcunda9 | JF962115 |  |  |  |  |
|  |  |  | 29 | PpraeKilcunda10 |  |  |  |  |  |
|  |  |  | 29 | PpraeKilcunda11 | JF962116 |  |  |  |  |
|  |  |  | 29 | PpraeKilcunda13 | JF962117 |  |  |  |  |
|  |  |  | 29 | PpraeKilcunda14 | JF962118 |  |  |  |  |
|  |  |  | 29 | PpraeKilcunda15 | JF962119 |  |  |  |  |
|  |  |  | 29 | PpraeKilcunda16 | JF962120 |  |  |  |  |
|  |  |  | 29 | PpraeKilcunda18 | JF962121 |  |  |  |  |
|  |  |  | 29 | PpraeKilcunda19 | JF962122 | JF962346 |  | JF961772 | JF961808 |
|  |  |  | 29 | PpraeKilcunda21 | JF962123 |  |  |  |  |
|  |  |  | 29 | PpraeKilcunda22 | JF962124 |  |  |  |  |
|  |  |  | 29 | PpraeKilcunda23 | JF962125 |  |  |  |  |
|  |  |  | 29 | PpraeKilcunda24 | JF962126 |  |  |  |  |
|  |  |  | 29 | PpraeKilcunda25 | JF962127 |  |  |  |  |
|  |  |  | 29 | PpraeKilcunda26 | JF962128 |  |  |  |  |
|  |  |  | 29 | PpraeKilcunda27 | JF962129 |  |  |  |  |
|  |  |  | 29 | PpraeKilcunda28 | JF962130 |  |  |  |  |
|  |  |  | 29 | PpraeKilcunda29 | JF962131 |  |  |  |  |
|  |  |  | 29 | PpraeKilcunda30 | JF962132 |  |  |  |  |
|  |  |  | 29 | PpraeKilcunda32 | JF962133 |  |  |  |  |
|  |  |  | 29 | PpraeKilcunda34 | JF962134 |  |  |  |  |
|  |  |  | 29 | PpraeKilcunda36 | JF962135 |  |  |  |  |
|  |  |  | 29 | PpraeKilcunda37 | JF962136 |  |  |  |  |
|  |  |  | 29 | PpraeKilcunda38 | JF962137 |  |  |  |  |
|  |  |  | 29 | PpraeWalkerville1 | JF962103 |  |  |  |  |
|  |  |  | 28 | PpraeWalkerville2 | JF962104 |  |  |  |  |
|  |  |  | 28 | PpraeWalkerville3 | JF962105 |  |  |  |  |
|  |  |  | 28 | PpraeWalkerville4 | JF962106 |  |  |  |  |
|  |  |  | 28 | PpraeWalkerville5 | JF962107 |  |  |  |  |
|  |  |  | 33 | PpraePortsea1 | JF962138 |  |  |  |  |
|  |  |  | 33 | PpraePortsea2 | JF962139 |  |  |  |  |
|  |  |  | 33 | PpraePortsea3 | JF962140 |  |  |  |  |
|  |  |  | 33 | PpraePortsea4 | JF962141 |  |  |  |  |
|  |  |  | 33 | PpraePortsea5 | JF962142 |  |  |  |  |
|  |  |  | 33 | PpraePortsea6 | JF962143 |  |  |  |  |
|  |  |  | 33 | PpraePortsea7 | JF962144 |  |  |  |  |
|  |  |  | 33 | PpraePortsea8 | JF962145 |  |  |  |  |
|  |  |  | 34 | PpraeMarengoBay1 | JF962146 |  |  |  |  |
|  |  |  | 34 | PpraeMarengoBay2 | JF962147 |  |  |  |  |
|  |  |  | 34 | PpraeMarengoBay3 | JF962148 |  |  |  |  |
|  | Chile |  | 50 | PpraeAntofagasta1 | JF962149 |  |  | JF961773 | JF961810 |
|  |  |  | 50 | PpraeAntofagasta2 | JF962150 | JF962347 |  | JF961774 | JF961811 |
|  |  |  | 50 | PpraeAntofagasta3 |  |  |  | JF961775 |  |
|  |  |  | 50 | PpraeAntofagasta4 |  |  |  |  |  |
|  |  |  | 50 | PpraeAntofagasta5 |  |  |  |  |  |
|  |  |  | 50 | PpraeAntofagasta6 | JF962151 |  |  |  |  |
|  |  |  | 50 | PpraeAntofagasta7 |  |  |  |  |  |
|  |  |  | 50 | PpraeAntofagasta9 |  |  |  |  |  |
|  |  |  | 50 | PpraeAntofagasta11 |  |  |  |  |  |
|  |  |  | 50 | PpraeAntofagasta12 |  |  |  |  | JF961812 |
|  |  |  | 50 | PpraeAntofagasta13 | JF962152 |  |  |  |  |
|  |  |  | 50 | PpraeAntofagasta14 | JF962153 |  |  |  |  |
|  |  |  | 50 | PpraeAntofagasta15 | JF962154 |  |  |  |  |
|  |  |  | 50 | PpraeAntofagasta16 | JF962155 |  |  |  |  |
|  |  |  | 50 | PpraeAntofagasta17 | JF962156 |  |  |  |  |
|  |  |  | 50 | PpraeAntofagasta18 |  |  |  |  |  |
|  |  |  | 50 | PpraeAntofagasta19 |  |  |  |  |  |
|  |  |  | 50 | PpraeAntofagasta20 |  |  |  |  |  |
|  |  |  | 50 | PpraeAntofagasta21 | JF962157 |  |  |  |  |
|  |  |  | 50 | PpraeAntofagasta22 |  |  |  |  |  |
|  |  |  | 50 | PpraeAntofagasta23 | JF962158 |  |  |  |  |
|  |  |  | 50 | PpraeAntofagasta24 | JF962159 |  |  |  |  |
|  |  |  | 50 | PpraeAntofagasta25 | JF962160 |  |  |  |  |
|  |  |  | 50 | PpraeAntofagasta26 |  |  |  |  |  |
|  |  |  | 50 | PpraeAntofagasta27 | JF962161 |  |  |  |  |
|  |  |  | 50 | PpraeAntofagasta28 | JF962162 |  |  |  |  |
|  |  |  | 50 | PpraeAntofagasta29 | JF962163 |  |  |  |  |
| Pyura sp. | Australia | Victoria | 26 | Pyura_spPortAlbert1 | JF962164 |  |  |  |  |
|  |  |  | 26 | Pyura_spPortAlbert2 | JF962165 |  |  |  |  |
|  |  |  | 26 | Pyura_spPortAlbert3 | JF962166 |  |  |  |  |
|  |  |  | 26 | Pyura_spPortAlbert4 | JF962167 |  |  |  |  |
|  |  |  | 26 | Pyura_spPortAlbert5 | JF962168 |  |  |  |  |
|  |  |  | 27 | Pyura_spPortWelshpool1 | JF962169 | JF962348 | JF962349 | JF961776 | JF961813 |
|  |  |  | 27 | Pyura_spPortWelshpool2 | JF962170 | JF962350 | JF962351 |  |  |
|  |  |  | 27 | Pyura_spPortWelshpool3 | JF962171 | JF962352 | JF962353 |  |  |
|  |  |  | 27 | Pyura_spPortWelshpool4 | JF962172 | JF962354 | JF962355 |  |  |
|  |  |  | 27 | Pyura_spPortWelshpool5 | JF962173 | JF962356 | JF962357 |  |  |
|  |  |  | 27 | Pyura_spPortWelshpool6 |  | JF962358 | JF962359 |  |  |
|  |  |  | 27 | Pyura_spPortWelshpool7 |  | JF962360 | JF962361 |  |  |
|  |  |  | 27 | Pyura_spPortWelshpool8 |  | JF962362 | JF962363 |  |  |
|  |  |  | 27 | Pyura_spPortWelshpool9 |  | JF962364 | JF962365 |  |  |
|  |  |  | 27 | Pyura_spPortWelshpool10 |  | JF962366 | JF962367 |  |  |
|  |  | Tasmania | 36 | Pyura_spBeautyPoint1 | JF962174 | JF962368 | JF962369 | JF961777 | JF961814 |
|  |  |  | 36 | Pyura_spBeautyPoint2 | JF962175 | JF962370 | JF962371 | JF961778 | JF961815 |
|  |  |  | 36 | Pyura_spBeautyPoint3 |  | JF962372 | JF962373 | JF961779 | JF961816 |
|  |  |  | 36 | Pyura_spBeautyPoint4 |  | JF962374 | JF962375 |  |  |
|  |  |  | 36 | Pyura_spBeautyPoint6 | JF962176 |  |  |  |  |
|  |  |  | 36 | Pyura_spBeautyPoint7 | JF962177 | JF962376 | JF962377 |  |  |
|  |  |  | 36 | Pyura_spBeautyPoint8 | JF962178 | JF962378 | JF962379 |  |  |
|  |  |  | 36 | Pyura_spBeautyPoint10 | JF962179 |  |  |  |  |
|  |  |  | 36 | Pyura_spBeautyPoint11 | JF962180 |  |  |  |  |
|  |  |  | 36 | Pyura_spBeautyPoint12 | JF962181 |  |  |  |  |
|  |  |  | 36 | Pyura_spBeautyPoint14 |  | JF962380 | JF962381 |  |  |
|  |  |  | 36 | Pyura_spBeautyPoint18 |  | JF962382 | JF962383 |  |  |
|  |  |  | 36 | Pyura_spBeautyPoint23 |  | JF962384 | JF962385 |  |  |
|  |  |  | 36 | Pyura_spBeautyPoint24 |  | JF962386 | JF962387 |  |  |
|  |  |  | 36 | Pyura_spBeautyPoint25 |  | JF962388 | JF962389 |  |  |
|  |  |  | 37 | Pyura_spTwoTreePoint1 | JF962182 |  |  |  |  |
|  |  |  | 37 | Pyura_spTwoTreePoint2 | JF962183 |  |  |  |  |
|  |  |  | 37 | Pyura_spTwoTreePoint3 |  | JF962390 | JF962391 |  |  |
|  |  |  | 37 | Pyura_spTwoTreePoint6 |  | JF962392 | JF962393 |  |  |
|  |  |  | 37 | Pyura_spTwoTreePoint7 |  | JF962396 | JF962397 |  |  |
|  |  |  | 37 | Pyura_spTwoTreePoint9 |  | JF962394 | JF962395 |  |  |
|  |  |  | 38 | Pyura_spTaroonaBeach320 |  | JF962398 | JF962399 |  |  |
|  |  |  | 38 | Pyura_spTaroonaBeach323 |  | JF962400 | JF962401 |  |  |
|  |  | South Australia | 39 | Pyura_spHenleyBeach1 | JF962184 | JF962402 | JF962403 |  |  |
|  |  |  | 39 | Pyura_spHenleyBeach2 | JF962185 | JF962404 | JF962405 |  |  |
|  |  |  | 39 | Pyura_spHenleyBeach3 | JF962186 | JF962406 | JF962407 |  |  |
|  |  |  | 39 | Pyura_spHenleyBeach4 | JF962187 |  |  |  |  |
|  |  |  | 39 | Pyura_spHenleyBeach5 | JF962188 |  |  |  |  |
|  |  |  | 39 | Pyura_spHenleyBeach9 |  |  |  | JF961780 | JF961817 |
|  |  |  | 39 | Pyura_spHenleyBeach10 | JF962189 |  |  | JF961781 | JF961819 |
|  |  |  | 39 | Pyura_spHenleyBeach12 | JF962190 |  |  |  |  |
|  |  |  | 39 | Pyura_spHenleyBeach13 | JF962191 |  |  | JF961782 | JF961818 |
|  |  |  | 40 | Pyura_spLargsBay1 | JF962192 |  |  |  |  |
|  |  |  | 40 | Pyura_spLargsBay2 | JF962193 |  |  |  |  |
|  |  |  | 40 | Pyura_spLargsBay3 | JF962194 |  |  |  |  |
|  |  |  | 40 | Pyura_spLargsBay4 | JF962195 |  |  |  |  |
|  |  |  | 41 | Pyura_spBrightonBeach1 | JF962196 |  |  |  |  |
|  |  |  | 41 | Pyura_spBrightonBeach2 | JF962197 |  |  |  |  |
|  |  |  | 41 | Pyura_spBrightonBeach3 | JF962198 |  |  |  |  |
|  |  |  | 41 | Pyura_spBrightonBeach4 | JF962199 |  |  |  |  |
|  | New Zealand |  | 43 | Pyura_spNewZealand |  | JF962408 | JF962409 |  | JF961820 |
|  |  |  | 43 | Pyura_spNewZealand |  | JF962408 | JF962409 |  | JF961821 |
|  |  |  | 43 | Pyura_spNewZealand |  | JF962408 | JF962409 |  | JF961822 |
|  |  |  | 43 | Pyura_spNewZealand |  | JF962408 | JF962409 |  |  |
|  |  |  | 44 | Pyura_spNewZealand |  | JF962408 | JF962409 |  |  |
|  |  |  | 44 | Pyura_spNewZealand |  | JF962408 | JF962409 |  |  |
|  |  |  | 44 | Pyura_spNewZealand |  | JF962408 | JF962409 |  |  |
|  |  |  | 45 | Pyura_spNewZealand |  | JF962408 | JF962409 |  |  |
|  |  |  | 45 | Pyura_spNewZealand |  | JF962408 | JF962409 |  |  |
|  |  |  | 45 | Pyura_spNewZealand |  | JF962408 | JF962409 |  |  |
|  |  |  | 45 | Pyura_spNewZealand |  | JF962408 | JF962409 |  |  |
|  |  |  | 45 | Pyura_spNewZealand |  | JF962408 | JF962409 |  |  |
|  |  |  | 45 | Pyura_spNewZealand |  | JF962408 | JF962409 |  |  |
|  |  |  | 45 | Pyura_spNewZealand |  | JF962408 | JF962409 |  |  |
|  |  |  | 45 | Pyura_spNewZealand |  | JF962408 | JF962409 |  |  |
|  |  |  | 45 | Pyura_spNewZealand |  | JF962408 | JF962409 |  |  |
|  |  |  | 45 | Pyura_spNewZealand |  | JF962408 | JF962409 |  |  |
|  |  |  | 46 | Pyura_spNewZealand |  | JF962408 | JF962409 |  |  |
|  |  |  | 46 | Pyura_spNewZealand |  | JF962408 | JF962409 |  |  |
|  |  |  | 46 | Pyura_spNewZealand |  | JF962408 | JF962409 |  |  |
|  |  |  | 46 | Pyura_spNewZealand |  | JF962408 | JF962409 |  |  |
|  |  |  | 46 | Pyura_spNewZealand |  | JF962408 | JF962409 |  |  |
|  |  |  | 46 | Pyura_spNewZealand |  | JF962408 | JF962409 |  |  |
|  |  |  | 46 | Pyura_spNewZealand |  | JF962408 | JF962409 |  |  |
|  |  |  | 46 | Pyura_spNewZealand |  | JF962408 | JF962409 |  |  |
|  |  |  | 46 | Pyura_spNewZealand |  | JF962408 | JF962409 |  |  |
|  |  |  | 46 | Pyura_spNewZealand |  | JF962408 | JF962409 |  |  |
|  |  |  | 47 | Pyura_spNewZealand |  | JF962408 | JF962409 |  |  |
|  |  |  | 47 | Pyura_spNewZealand |  | JF962408 | JF962409 |  |  |
|  |  |  | 47 | Pyura_spNewZealand |  | JF962408 | JF962409 |  |  |
|  |  |  | 47 | Pyura_spNewZealand |  | JF962408 | JF962409 |  |  |
|  |  |  | 47 | Pyura_spNewZealand |  | JF962408 | JF962409 |  |  |
|  |  |  | 47 | Pyura_spNewZealand |  | JF962408 | JF962409 |  |  |
|  |  |  | 47 | Pyura_spNewZealand |  | JF962408 | JF962409 |  |  |
|  |  |  | 47 | Pyura_spNewZealand |  | JF962408 | JF962409 |  |  |
|  |  |  | 47 | Pyura_spNewZealand |  | JF962408 | JF962409 |  |  |
|  |  |  | 47 | Pyura_spNewZealand |  | JF962408 | JF962409 |  |  |
|  |  |  | 48 | Pyura_spNewZealand |  | JF962408 | JF962409 |  |  |
|  |  |  | 48 | Pyura_spNewZealand |  | JF962408 | JF962409 |  |  |
|  |  |  | 48 | Pyura_spNewZealand |  | JF962408 | JF962409 |  |  |
|  |  |  | 49 | Pyura_spNewZealand |  | JF962408 | JF962409 |  |  |
|  |  |  | 49 | Pyura_spNewZealand |  | JF962408 | JF962409 |  |  |
| Pyura dalbyi | Australia | Victoria | 27 | PdalbPortWelshpool1 | JF962200 |  |  |  |  |
|  |  |  | 27 | PdalbPortWelshpool2 | JF962201 |  |  |  |  |
|  |  |  | 27 | PdalbPortWelshpool3 | JF962202 |  |  |  |  |
|  |  |  | 30 | PdalbStonyPoint1 | JF962203 |  |  |  |  |
|  |  |  | 30 | PdalbStonyPoint2 | JF962204 |  |  |  |  |
|  |  |  | 30 | PdalbStonyPoint3 | JF962205 |  |  |  |  |
|  |  |  | 30 | PdalbStonyPoint4 | JF962206 |  |  |  |  |
|  |  |  | 31 | PdalbHastings1 | JF962207 |  |  |  |  |
|  |  |  | 31 | PdalbHastings2 | JF962208 |  |  |  |  |
|  |  |  | 31 | PdalbHastings3 | JF962209 |  |  |  |  |
|  |  |  | 31 | PdalbHastings4 | JF962210 |  |  |  |  |
|  |  |  | 31 | PdalbHastings5 | JF962211 |  |  |  |  |
|  |  |  | 32 | PdalbMornington1 | JF962212 |  |  |  |  |
|  |  |  | 32 | PdalbMornington2 | JF962213 |  |  |  |  |
|  |  |  | 32 | PdalbMornington3 | JF962214 |  |  |  |  |
|  |  |  | 35 | PdalbPortarlington1 | JF962215 |  |  |  |  |
|  |  |  | 35 | PdalbPortarlington2 | JF962216 |  |  |  |  |
|  |  |  | 35 | PdalbPortarlington3 | JF962217 |  |  |  |  |
|  |  |  | 35 | PdalbPortarlington4 | JF962218 |  |  |  |  |
|  |  | Western Australia | 42 | PdalbAlbany1 | JF962219 |  |  |  |  |
|  |  |  | 42 | PdalbAlbany2 | JF962220 |  |  |  |  |
|  |  |  | 42 | PdalbAlbany3 | JF962221 |  |  |  |  |
|  |  |  | 42 | PdalbAlbany4 | JF962222 |  |  |  |  |
|  |  |  | 42 | PdalbAlbany5 | JF962223 | JF962410 | JF962411 | JF961783 | JF961823 |
|  |  |  | 42 | PdalbAlbany8 | JF962224 | JF962412 | JF962413 | JF961784 | JF961824 |
|  |  |  | 42 | PdalbAlbany10 | JF962225 |  |  |  |  |
|  |  |  | 42 | PdalbAlbany11 | JF962226 |  |  |  |  |
|  |  |  | 42 | PdalbAlbany12 | JF962227 |  |  |  |  |
|  |  |  | 42 | PdalbAlbany13 | JF962228 | JF962414 | JF962415 | JF961785 | JF961825 |
|  |  |  |  |  |  |  |  |  |  |
| Pyura spinifera | Australia | New South Wales |  | PspinNewcastle |  |  | JF962230 |  | JF961826 |
| Pyura dura |  |  |  | Pdura |  |  |  | JF961786 |  |
| Styela plicata | Australia | Western Australia |  | StyelaWA1 |  |  | JF962232 |  |  |
| Botrylloides leachi | Australia | New South Wales |  | BleachiJulianRocks5 |  |  | JF962229 |  |  |
| Botryllus magnicoecus | Australia | New South Wales |  | BmagnSydney1 |  |  | JF962231 |  |  |
|  |  |  |  |  |  |  |  |  |  |
|  |  |  |  |  |  |  |  |  |  |
